# Investigation of viscoelastic behaviour of rice-field bean gluten-free dough using the biophysical characterization of proteins and starch: a FT-IR study

**DOI:** 10.1007/s13197-019-03602-2

**Published:** 2019-02-22

**Authors:** Awatif Fetouhi, Leila Benatallah, Agnieszka Nawrocka, Monika Szymańska-Chargot, Abdallah Bouasla, Marta Tomczyńska-Mleko, Mohammed Nasreddine Zidoune, Agnieszka Sujak

**Affiliations:** 1 Institut de la Nutrition, de l’ Alimentation et des Technologies Agro-Alimentaires I.N.A.T.A.A., Université des Frères Mentouri, Constantine 1, Route de Ain El_Bey, Constantine, Algeria; 2 Institute of Agrophysics, Polish Academy of Sciences, Doświadczalna 4, 20-290 Lublin, Poland; 3 Institute of Plant Genetics, Breeding and Biotechnology, Faculty of Agrobioengineering, University of Life Sciences in Lublin, Akademicka 15, 20-950 Lublin, Poland; 4 Department of Biophysics, University of Life Sciences in Lublin, Akademicka 13, 20-933 Lublin, Poland

**Keywords:** FT-IR, Gluten-free dough, Protein structure, Rheological behaviour, Starch reorganization

## Abstract

Gluten-free bread making success is closely linked to the biophysical behaviour of dough. Quality of these doughs is largely determined by the properties of their proteins and starch. This study aimed to explain, at the structural level the rheological behaviour of gluten-free rice-field bean dough compared to that of soft wheat. The conformational aspects of proteins and starch were studied using Fourier transformed infrared spectroscopy (FT-IR). Doughs of soft wheat, rice, field bean, mixture of rice-field bean flour and the same mixture where a portion of rice flour underwent hydrothermal treatment were studied. The results show that viscous and viscoelastic components of gluten-free doughs were changed by supplementation of rice with field bean flour. Most of gluten-free doughs possessed a higher storage modulus in comparison with soft wheat dough. Analysis of FT-IR spectra in the amide I region conveyed to find the differences relative to soft wheat flour dough showed that in non-gluten doughs the increase in β-sheet content was observed at the expense of β-turns. These results were confirmed by amide I deconvolution. Gluten-free doughs contained more β-sheet structure as compared to soft wheat dough and less β-turns inducing high structuralization level that characterized this type of dough matrix. Concerning starch, the supplementation with rice-field bean generated the reorganization of field bean and rice doughs starches approaching that of wheat dough.

**Electronic supplementary material:**

The online version of this article (10.1007/s13197-019-03602-2) contains supplementary material, which is available to authorized users.

## Introduction

Gluten-free baking is a big challenge for food technologists and cereal researchers. The secret of wheat bread dough quality lies within the unique properties of gluten proteins. Under the effect of hydration and the energy provided by kneading, gliadins and glutenins present in the dough form a continuous viscoelastic network. The presence of gluten is closely related to the dough and bread quality (Sivam et al. 2013). Gluten-free bread doughs showed various differences in rheological properties compared to those of soft wheat. They displayed much less cohesiveness and elasticity than wheat dough. They were smoother, stickier, weakly elastic and pasty, with more similarity to cake dough (Cauvain 1998). Differences in the rheological properties were closely related to the absence of gluten, which might result from the inability of gluten-free ingredient proteins to form viscoelastic network similar to that of gluten (Houben et al. 2012). The improvement of gluten-free formulas was also an objective of the previous studies in order to mimic gluten network by several types of improvers (Benatallah et al. 2012; Bourekoua et al. 2016; Lazaridou et al. 2007; Matos and Rosell 2012). All of these studies were based on the rheological behaviour of dough and technological properties of bread (bread volume and alveolar structure of crumb) in order to estimate and explain the bread quality features. The structural aspect of this type of dough remains poorly known and poorly studied to understand its low quality compared to that of soft wheat, on one hand. On the other hand, several studies have been conducted on the structure of protein fractions of gluten-free ingredients such as rice, maize, kidney bean and field pea (Ellepola et al. 2005; Mejia et al. 2007; Shevkani et al. 2015). The above studies aimed to explain the functionality of the protein fractions of these grains in relation to the structural aspect. The structural explanation of the ability of these ingredients in the manufacture of complex foods (i.e. gluten free bread dough) remains poorly understood.

FT-IR is a spectroscopic technique that can be used to study the molecular structure of heterogeneous foods and biological systems. It is non-destructive and can be applied in studies on small amounts of dry and wet samples (Wang et al. 2015). This method has been already used to highlight the relationship between protein structure, rheological characteristics of doughs and technological properties of wheat bread (Sivam et al. 2013; Wang et al. 2015). It was also applied to study changes in the gluten structure as a result of supplementation of wheat dough with dietary fibre preparations (Nawrocka et al. 2017) and fibre polysaccharides (Nawrocka et al. 2018).

This study aimed to explain, at the structural level, the rheological behaviour of gluten-free rice-field bean dough compared to that of soft wheat. It includes a search for relations between rheological parameters, protein and starch structure assessed with means of FT-IR spectroscopic technique. This study also aimed to explain the effect of rice-field bean flour supplementation and hydrothermal treatment of rice on final dough structure.

## Materials and methods

### Materials

Wheat flour (*Triticum aestivum*) (MłynPiaski, Poland) was purchased from Lublin local market. Sodium chloride was purchased from Sigma Aldrich (Poland). Rice (*Oryza sativa*) (Indian Basmati brand) and field bean (*Vicia faba L.*) (Albehera, Egypt) seeds were milled using a MG E3 grinder (UMA Rouiba, Algeria). The ground material was sieved manually using a 200 μm sieve. Gluten-free formula was obtained by mixing of a mass ratio of 2/3 rice flour and 1/3 field bean flour in order to obtain balanced amounts of amino-acids (Benatallah et al. 2012).

## Methodology

### Doughs preparation

Wheat and gluten-free doughs (rice, field bean, rice/field bean formula and rice/field bean improved formula) were prepared using Farinograph-E equipped with a 50 g mixer (model 81,101,142, Brabender, Germany). 50 g of each type of flour and aqueous solution of sodium chloride (2%, w/w) were kneaded for 20 min with an appropriate amount of distilled water according to the Farinograph optimal conditions [500 Farinograph unit (FU)]. The components were mixed as follows: wheat dough − 50 g of wheat flour + 25.9 mL of water, rice dough − 50 g of rice flour + 46.5 mL of water, field bean dough − 50 g of field bean flour + 22.8 mL of water, formula dough − 33.33 g of rice flour + 16.67 g of field bean flour + 39 mL of water, improved formula dough − 26.43 g of rice flour, 16.67 g of field bean flour + 6.9 g of rice flour that underwent hydrothermal treatment + 19 mL of water. Hydrothermal treatment was conducted as follows: 34.5 mL of distilled water was added to 6.9 g of rice flour and the mixture was heated until reaching the temperature of 65 °C according to Bourekoua et al. (2016).

In order to allow the relaxation process after mixing, doughs were subjected to a rest period of 20 min at room temperature (Benatallah et al. 2012). All samples were then lyophilized for 24 h (0.04 mbar [0.04 hPa], − 50 °C). After freeze-drying, doughs were milled to powder in laboratory grinder (Optimum RK-0150, Germany) and used in the FT-IR analysis. Before FT-IR measurement, the samples were moisturized with 10% aqueous solution of deuterium oxide (D_2_O) for 2 h. The purpose of using of D_2_O was to eliminate the overlap effect of the water bands on amide I band (Kong and Yu 2007).

### Rheological characterization of doughs

#### Farinograph parameters

Water absorption (WA) (%) and time of dough development (TDD) (min) at 500 FU dough consistency (standard procedure ICC 115/1) were obtained from farinograph.

#### Small deformation mechanical test: oscillatory test

Small deformation mechanical test was conducted in order to estimate the differences in viscoelastic behaviour of gluten-free doughs in comparison with soft wheat (control). Oscillatory test was done using the Rheo-Stress 300 rheometer (Karlsruhe, Germany) equipped with parallel plates of 5 cm in diameter with 2 mm adjusted gap, according to the method described by Ronda et al. (2013). The rested dough (20 min) was placed between the plates and subjected to scanning with a frequency sweep ranging from 0.1 to 10 Hz and low strain value (0.1%) in order to keep examined doughs in linear viscoelastic region determined in strain sweep tests performed at 1 Hz frequency. Before the measurement, the excess dough was removed. The measurements were performed at 20 ± 0.01 °C. The storage (G′) modulus, loss (G″) modulus and the loss tangent (tan δ) were recorded. For each type of dough, the test was done in duplicate.

### Fourier transformed infrared (FT-IR) spectra collection and analysis

Nicolet 6700 FTIR spectrometer (Thermo Scientific, Madison, WI, USA) equipped with a diamond attenuated total reflectance attachment (ATR) was used to collect spectra between 4000 and 400 cm^−1^ at a 4 cm^−1^ resolution. To obtain an optimal signal-to-noise ratio, 128 scans were collected. Each spectrum was baseline-corrected using OMNIC software (version 8.2, Thermo Fischer Scientific Inc., Madison, WI, USA). The analysed spectra were averaged over five registered spectra series. In order to eliminate the effect of water bands from samples, the spectrum of 10% D_2_O aqueous solution was subtracted from all spectra according to Nawrocka et al. (2017). In order to separate the overlapping bands in the amide I region and to allow an identification of the constitutes of protein secondary structure, the second derivative of the amide I band was calculated using five points two-degree polynomial function. Following that the second derivative spectra were smoothed with 11-points, two-degree polynomial Savitsky–Goly function according to Seabourn et al. (2008) and Susi and Byler (1983). To estimate quantitatively the fraction of each type of secondary structure amide I band (1590-1720 cm^−1^) was deconvoluted with Gaussian curves using Grams 32 AI (version 9) software (Galactic, USA) according to the second derivative peak identification. The quality of the band deconvolution was indicated by the following parameters: R^2^ > 0.99, solution converged and χ^2^ < 0.001. The relative composition of secondary protein structures participating in the amide I band was expressed as percentage of the area of the fitted region expressed as a relative area of components centred at specific wavenumbers (Bock and Damodaran 2013). The secondary structure assignment was based on absorption wavenumbers according to the studies of Bock and Damodaran (2013) and Yang et al. (2015).

To highlight the differences in secondary structures (amide I band) between protein backbone of gluten-free doughs and that of soft wheat dough (control), the subtraction of soft wheat dough spectrum from those of gluten free doughs was done. To estimate the changes in secondary structure of proteins induced by rice-field bean supplementation, the spectra of field bean and formula doughs were subtracted from that of rice dough. Finally, to analyse the effect of hydrothermal treatment of rice flour, the spectrum of formula dough was subtracted from that of the improved formula. The subtraction within amide I bands was performed using ORIGIN pro 8 SR0 (v.8.0724, origin lab corporation, USA). Before the subtraction, all amide I bands were baseline corrected and area normalized. Crystalline and amorphous fractions of starch were studied by estimation of intensity ratio I (1047 cm^−1^)/I (1022 cm^−1^) (Smits et al. 1998).

### Statistical analysis

Variance analysis (ANOVA) and significant difference test (Tukey (HSD) test) were used for farinograph (WA, TDD) and rheological parameters (G′, G″ and tan δ). Principal component analysis (PCA) was used to highlight the relationship between rheological properties and protein structure as well as starch reorganization characteristics in order to explain the rheological behaviour of gluten-free bread dough versus that of soft wheat. Both statistical tests were performed using the XLSTAT software (version 2009.1.01, Addinsoft) at a significance level of α = 0.05. Results were expressed as mean ± standard deviation.

## Results and discussion

### Rheological properties

Table 1 summarizes farinograph (WA, TDD) and rheological parameters (G′, G″ and tan δ) in the linear viscoelastic domain (strain 0.1%) at 1 Hz frequency.

**Table 1:** Farinograph (WA, TDD) characteristics and rheological parameters (G’, G” and tan δ) in the linear viscoelastic domain (strain 0.1%) at 1 Hz frequency

Doughs	WA (%)	TDD (min)	G′ (Pa)	G″ (Pa)	tan δ
Wheat	52.5 ± 0.201 c	1.6 ± 0.1 c	32,940 ± 689 c	14,780 ± 309 b	0.449 ± 0.009 a
Rice	94 ± 0.203 a	9.85 ± 0.1 a	55,330 ± 1157 b	8523 ± 178 c	0.154 ± 0.003 d
Field bean	45.6 ± 0.201d	8.21 ± 0.1 b	58,010 ± 1213 b	26,350 ± 551 a	0.454 ± 0.009 a
Formula	79 ± 0.200 b	8.07 ± 0.1 b	51,075 ± 1068 a	14,165 ± 92 b	0.277 ± 0.004 c
Improved formula	38.5 ± 0.204 e	7.78 ± 0.1 b	11,920 ± 1202 d	2920 ± 87 d	0.254 ± 0.018 b

WA—water absorption and DDT—time of dough development were obtained from doughs farinogramm. Storage modulus (G′), loss modulus (G″) and loss tangent (tan δ) were collected from rheometer experiment in viscoelastic linear region

Different letters following the values in the columns indicate a significant difference (*P *< 0.05)

Rice dough had the highest water absorption and development time values which were respectively 1.79 and 6.15 times higher (*P *< 0.05) than those of soft wheat dough. The high water absorption can be explained by the hydrophilic nature of rice flour. According to long time of dough development, rice dough was characterized by a poor quality for baking, a limited machinability and relaxing stretchable properties (Lazaridou et al. 2007; Sivaramakrishnan et al. 2004). Rice-field bean supplementation and hydrothermal treatment of rice led to a significant (*P *< 0.05) decrease in water absorption and development time of rice dough reaching the values characteristic of soft wheat. Substitution of 1/3 fraction of rice flour with field bean flour (formula) characterized by a low WA and TDD, led to the decrease of the hydrophilic character (decrease of rice amount) and increased machinability. In addition to the effect of the substitution, the hydrothermal treatment of 13.8% of rice fraction constituting the formula modified the hygroscopic properties of rice flour by altering the functional properties of its constituents. Results obtained from farinograph for all doughs indicated that soft wheat dough showed better quality compared to gluten-free dough because it had the lowest development time (Lazaridou et al. 2007). The low WA of improved formula dough was probably due to the additional amount of water provided by the hydrothermally treated rice flour fraction since it was added to the formula flour in the form of a gel at the beginning of mixing.

The storage modulus (G′) represents the energy stored by the system after deformation which could be released (elastic deformation). The loss modulus (G″) represents the dissipated energy, which is lost as inner friction (viscous deformation). Tan δ indicates the relative contribution of the viscous and elastic components in material behaviour (Dus and Kokini 1990). Tan δ is less than 1 if the sample is more like an elastic material. When tan δ is greater than 1, the loss modulus is predominant and the sample behaves like a viscous material (Khatkar and Schofield 2002). The values of tan δ lower than 1 for all investigated doughs indicate that all of them behave more like elastic, solid material. In our study, tan δ of all the examined doughs decreased with increased frequency up to the value of 0.07 Hz and then an increase in tan δ was noted. Increased frequency up to 0.07 Hz caused a shorter time for relaxation of the dough material and it behaved more elastically with decreased values of tan δ. At the higher frequency more energy was introduced into the samples, which could break some of the weaker bonds in the dough structure. It was manifested by an increase in tan δ. The frequency sweep of the moduli showed that their values increased in the whole frequency range. It means that, generally, the samples were strong gels and no major breaking of the microstructure happened under sinusoidal dynamic deformations.

**Fig. 1:**
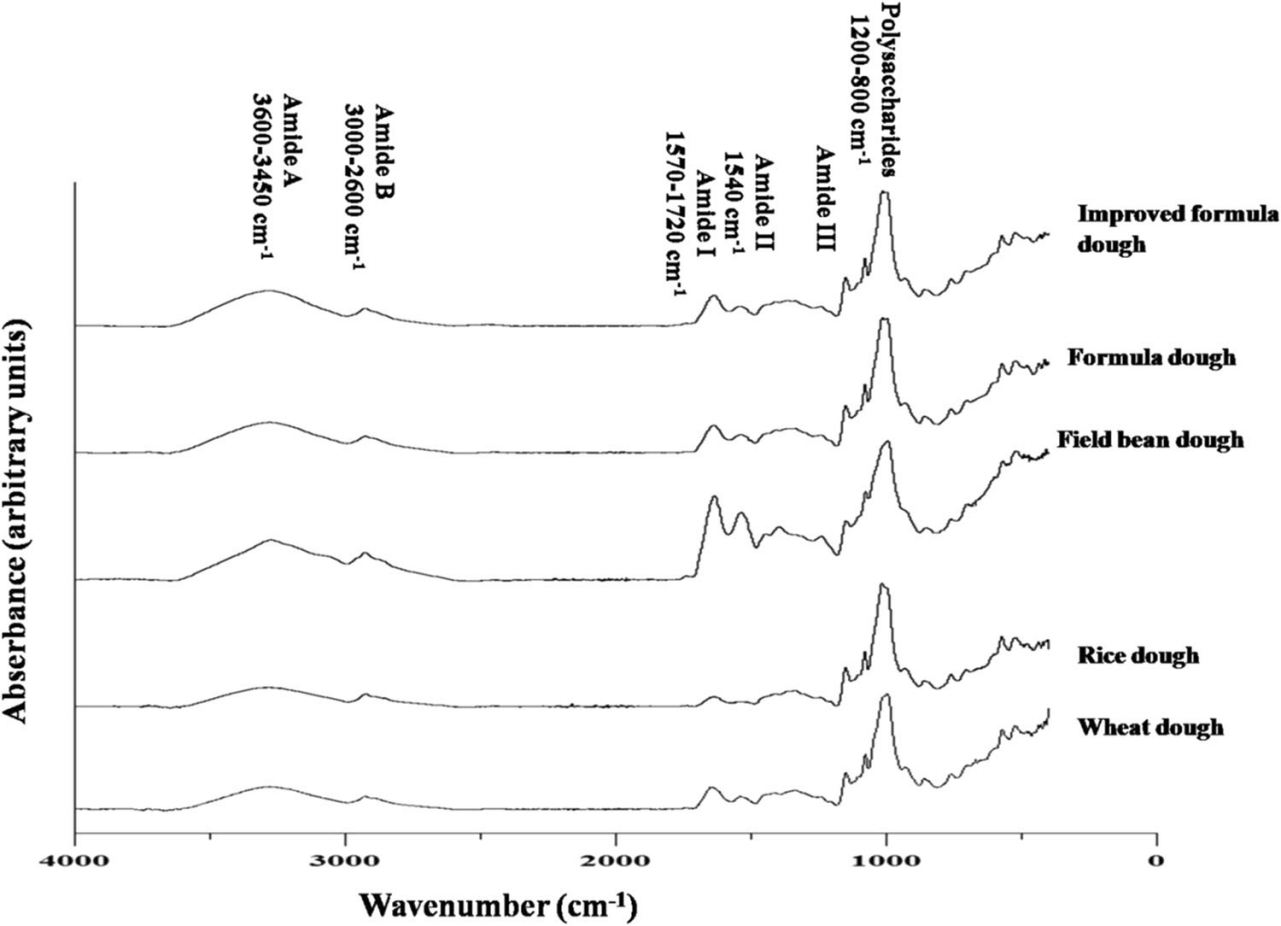
Baseline-corrected FTIR spectra of examined doughs (as indicated). Characteristic bands: NH stretching vibration of amide A (3600–3450 cm^−1^) and amide B (3000–2600 cm^−1^) bands. Peaks centred at 1640 cm^−1^ (amide I) and 1540 cm^−1^ (amide II) due to CO carbonyl stretch with minor contribution from out of plane CN stretching vibration and NH bonding and CH stretching of proteins, respectively. Band between 1350 cm^−1^ and 1200 cm^−1^, characteristic of amide III region, represents NH in-plane bending coupled with C–N-stretching and also includes CH and NH deformation modes. Spectral region of polysaccharides (800–1200 cm^−1^) is characteristic for backbone vibrations of CO, CN and CC bonds

A similar behaviour of rice-based gluten-free doughs was observed by Lazaridou et al. (2007) and Weipert (1990). The curves representing rheological parameters as a function of frequency were present in supplementary material (Fig. 1S).

The obtained results show that at the frequency of 1 Hz, all doughs give the storage modulus higher than the loss modulus and tan δ values less than 1. Field bean dough showed the highest storage modulus (G′ = 58,010 Pa) while that of the improved formula was the lowest (G′ = 11,920 Pa). Rice and field bean doughs gave significantly much higher values of G′ than that of the soft wheat dough. Only the improved formula dough showed a lower G′ value than the wheat dough (32,940 Pa). According to Lazaridou et al. (2007), the highest values of G′ that characterize rice, field bean and formula doughs demonstrate that these gluten-free doughs were more stiff and less extensible than soft wheat dough. The above-mentioned authors indicate that the stiffness of gluten-free doughs is affected by the level of the dough matrix structuralization and that the G′ value increases with increasing of the structuralization level. He and Hoseney (1992) suggested that the low value of G′ was due to the existence of probably significant interactions between the proteins and the other components of dough. This may indicate that gluten-free proteins show low ability to interact with other dough constituents in comparison with gluten proteins, which may explain the low elasticity of gluten-free matrices.

Field bean dough had higher loss modulus (G″) than that of soft wheat. As stated by Caballero et al. (2016), increase in storage (G′) and loss (G″) moduli can serve as the confirmation of strengthening action of additives due to crosslinking effect on different flour protein fraction. The above authors suggest that the structure of flour proteins could be changed due to the formation of large insoluble polymers. The lowest G″ was that of improved formula dough and it was 5.06 times lower than that of soft wheat. Supplementation of rice flour with field bean significantly lowered the loss modulus of formula dough, which was similar to that of the wheat dough. The similar value of loss modulus to that of soft wheat dough presented by formula dough indicated that supplementation affects viscous behaviour rather than elastic behaviour.

Rice, formula and improved formula doughs gave tan δ values lower than those of field bean and soft wheat doughs. The lowest tan δ was recorded for the rice dough and was 2.91 times lower than that of the soft wheat dough, which shows that this type of dough behaves more like a solid material. According to Gujral et al. (2003), the increase in the tan δ value explains the decrease in the relative contribution of a solid character in dough formation. This could explain that the low tan δ value of gluten-free doughs is due to the strong participation of solid character in gluten-free dough formation than to the viscous one. Weipert (1990) has shown that dough with a low tan δ reflects a rigid and stiff material having less elastic texture and dry surface appearance. In the study presented here it was found that the supplementation of rice flour with field bean flour changes its viscoelastic behaviour, whereas the hydrothermal treatment of rice does not have any effect.

### FT-IR spectroscopy

Figure 1 shows FT-IR spectra of freeze-dried wheat and gluten-free doughs. The recorded spectra show strong bands between 3600 and 2600 cm^−1^ comprising mainly amide A (3600–3450 cm^−1^) and Amide B (3000–2600 cm^−1^) resulting from NH stretching vibration. (van Velzen et al. 2003; Sivam et al. 2013). Typical protein bands of amide I and amide II have peaks centred at ~ 1640 cm^−1^ and ~ 1540 cm^−1^ respectively (due to CO carbonyl stretch with minor contribution from out of plane CN stretching vibration and NH bonding and CH stretching of proteins, respectively) (van Velzen et al. 2003). Amide III band between 1350 cm^−1^ and 1200 cm^−1^ represents NH in-plane bending coupled with C–N-stretching and also includes CH and NH deformation modes (Meziani et al. 2011; Sivam et al. 2013). A maximum absorbance is noted at ~ 1334 cm^−1^ for soft wheat, rice, formula and improved formula spectra and at ~ 1390 cm^−1^ for field bean doughs.

Another spectral region (800–1200 cm^−1^) is characteristic for backbone vibrations of CO, CN and CC bonds frequently assigned to polysaccharides (Sivam et al. 2013). Starch and proteins are the major components of examined flours and have a fundamental role in dough production, which is why the amide I and starch bands are examined in detail in order to understand their role.

### Analysis of the secondary structure of proteins

**Fig. 2:**
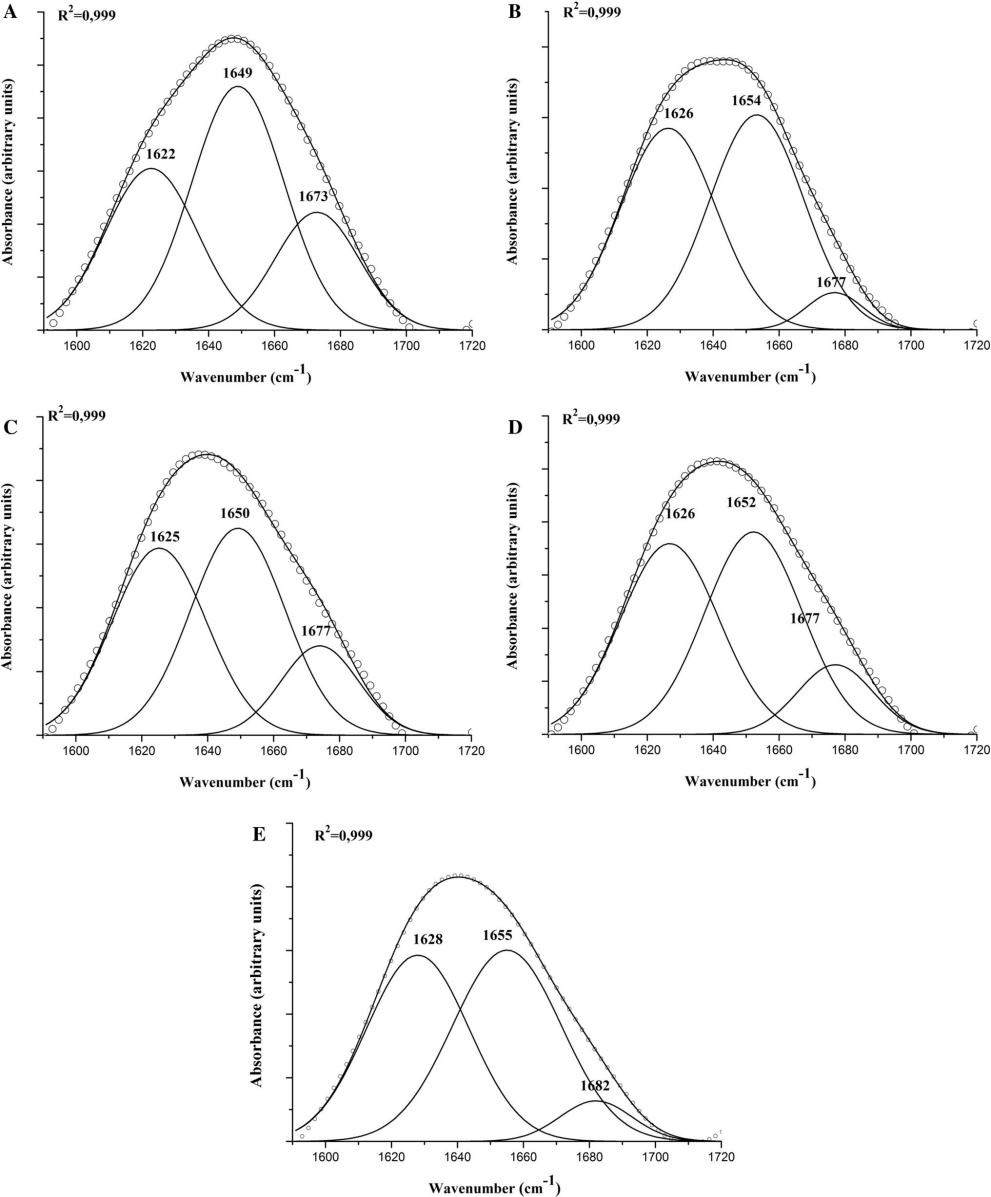
Deconvoluted amide I band (1590–1720 cm^−1^) of doughs; **A** soft wheat dough, **B** rice dough, **C** field bean dough, **D** formula rice-filed bean dough and **E** improved formula dough. Solid line: fitted curve, open circles: original data. Curve fitting was conducted using Gaussian–Lorentzian mix function

The second derivative of FT-IR spectra of dough in amide I region (1590–1720 cm^−1^) is shown on Fig. 2S (Supplementary materials).

The second derivatives of FT-IR spectra in amide I region show the presence of three characteristic areas for all examined doughs. The areas between 1620 and 1644 cm^−1^ are assigned to β-sheet structure (Bock and Damodaran 2013; Kong and Yu 2007). The following bands are visible: bands centred at 1619, 1627 cm^−1^ for wheat dough, at 1621 and 1631 cm^−1^ for the rice dough, at 1631 cm^−1^ for field bean dough, at 1623, 1631 cm^−1^ for formula dough and at 1632 cm^−1^ for improved formula dough. Another spectral region between 1649 and 1659 cm^−1^ represents α-helix structure (Van Velzen et al. 2003). In that case the following bands are observed: bands centered at 1651 cm^−1^ for wheat dough, at 1653 cm^−1^ for the rice dough, at 1655 cm^−1^ for field bean dough, at 1654 cm^−1^ for formula dough and at 1654 cm^−1^ for improved formula dough. β-turn structure is represented in the spectral region between 1660 and 1688 cm^−1^ (Kong and Yu 2007; Yang et al. 2015). Bands centred at 1674 cm^−1^ for wheat dough, at 1674 cm^−1^ for rice dough, at 1685 cm^−1^ for field bean dough, at 1683 cm^−1^ for formula dough and at 1686 cm^−1^ for improved formula dough can be observed. No spectral features indicating the presence of random structures for all types of dough and no bands were found in the region between 1590 and 1619 cm^−1^ (Kong and Yu 2007). On the other hand, the broad peak assigned to β-sheet structure shown by the second derivative of gluten-free doughs spectra, compared to that of soft wheat, indicates the high involvement of this type of secondary structure in the formation of gluten-free proteins network (Susi and Byler 1983).

To estimate the quantitative participation of each type of secondary structure in the doughs’ protein network, curve fitting was proceeded. Figure 2 shows deconvoluted amide I bands (1590–1720) cm^−1^ where the protein backbone of all the examined doughs was fitted with three peaks according to second derivative peak identification which was similar to that given by Nawrocka et al. (2017).

Protein network forming soft wheat dough can be characterized with peaks centered at 1622, 1649 and 1673 cm^−1^ (Fig. 2A) assigned to β-sheet, α-helix and β-turn, respectively (Bock and Damodaran 2013; Kong and Yu 2007; van Velzen et al. 2003; Yang et al. 2015). Quantitatively, amide I band shows dominance of α-helix structure (48%) followed by β-sheet (31%), then β-turn (21%) (Table 2). Our results are different from those found by other authors (Bock and Damodaran 2013; van Velzen et al. 2003) who showed the dominance of β-sheet structure in pure gluten proteins. The same results as those presented by the above authors have been found by Sivam et al. (2013) who have investigated the effect of fibre and polyphenols-enriched flours on bread polymers conformation. In our case one has to be aware of the presence of C=O stretching at ca. 1630 cm^−1^ which overlaps the β-sheet characteristic peak.

**Table 2:** Relative composition of secondary dough protein structures participating in the amide I band calculated on the basis of deconvolution of FT-IR spectra in the amide I region

	β-Sheet (%)	α-Helix (%)	β-Turn (%)
Wheat	31	48	21
Rice	46	50	4
Field bean	40	45	15
Formula	43	45	12
Improved formula	44	48	8

In the case of rice dough proteins (Fig. 2B) constituents, peaks are centred at 1626 cm^−1^, 1654 cm^−1^ and 1677 cm^−1^. Similarly to wheat, the protein network of rice dough is characterized by a dominance of α-helix (50%). It contains 46% of β-sheet structure and a small fraction (4%) assigned to β-turn structure (1677 cm^−1^) (Table 2) (Kong and Yu 2007; Yang et al. 2015). The same results were obtained by Ellepola et al. (2005) and Gorinstein et al. (1996) who showed the predominance of α-helix structure in rice globulin.

The amide I band of field bean dough (Fig. 2C) shows absorbance maxima at 1625, 1650 and 1674 cm^−1^. In that case a dominance of α-helix structure (45%) followed by β-sheet (40%) and β-turn (15%), which is quantitatively more close to that of wheat proteins than that of rice (Table 2) was observed. Unlike rice dough, the protein backbone of field bean dough shows a higher fraction of β-turn structure (1674 cm^−1^) with a lower percentage than that of the soft wheat dough (van Velzen et al. 2003; Yang et al. 2015). Previous studies on structural and functional characterization of kidneys and field pea protein isolates showed the predominance of β-sheet structure in the protein backbone (Shevkani et al. 2015). Structural characterization of globulin fraction for different mono- and dicotyledonous seeds conducted by Marcone et al. (1998) revealed that globulin fractions generally had high concentrations of β-sheet and lower levels of α-helix structures. All these studies showed a similar protein secondary structure to that obtained in our study for field bean dough.

The effect of supplementation of rice flour with field bean flour (formula) on proteins behaviour is shown on Fig. 2D. The supplementation of rice dough with field bean flour results in the slight increase in β-sheet structure (1626 cm^−1^-43%) accompanied with the decrease in β-turn structure (1677 cm^−1^-12%). In comparison with the control of soft wheat, the supplementation gives a protein network characterized by a higher fraction of β-sheet at the expense of β-turn structure (Fig. 2D and A, respectively). It produces the decrease in α-helix content of rice dough, approaching that of the wheat dough.

The amide I band of improved formula dough generally has a shape similar to that of formula dough (Fig. 2E). The deconvolution of this band shows similar fractions of α-helix (48%) and β-sheet structure (44%). Small fraction of β-turn structure (8%) is present in the composition of this band (Table 2). Peaks which represent β-sheet and β-turn are shifted respectively to 1628 cm^−1^ and 1682 cm^−1^ compared to those of formula dough. Shift in the position of absorbance maxima in case of improved formula can be explained by the effect of the hydrothermal treatment. The shift to the higher frequencies shows that the secondary structures which constitute the protein backbone of formula improved dough are characterized by shorter bonds and higher vibrational energy.

**Fig. 3:**
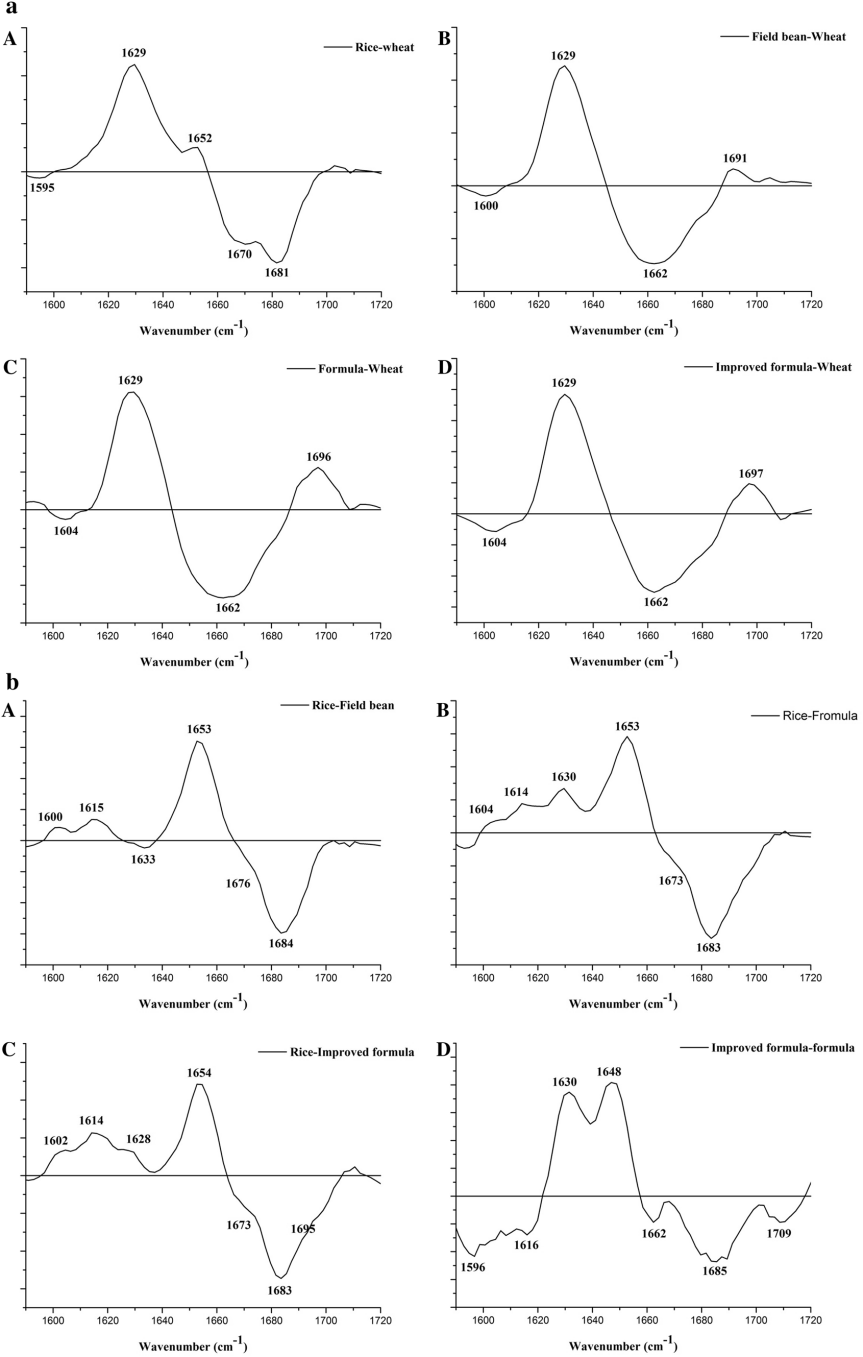
Amide I region differential spectra: **a** between rice (**A**), field bean (**B**), formula (**C**) and improved formula (**D**) doughs and wheat dough; **b** between rice and field bean (**A**), formula (**B**) and improved formula (**C**) doughs. D shows the difference spectra between amide I region of improved formula and formula dough **(b)**. Calculation of differences was performed after baseline correction and surface normalization of amide I bands

In our study, difference spectra between wheat and non-gluten doughs were analysed to confirm the secondary structure obtained by the deconvolution of amide I bands. As seen from Fig. 3a similar structural effects are observed for all non-gluten doughs. Generally, the increase in β-sheet content is observed at the expense of β-turns (positive peak at 1629 cm^−1^ versus negative band between 1658 and 1690 cm^−1^) (Bock and Damodaran 2013; Kong and Yu 2007; Yang et al. 2015). In the case of rice protein, peak centred at 1652 cm^−1^ shows the slight increase in α-helix content which confirms the analysis obtained by the spectra deconvolution. Broad negative band with two maxima centred at 1670 and 1681 cm^−1^ can be assigned to β-turns and anti-parallel β-sheet, respectively (Kong and Yu 2007; Yang et al. 2015). The last one (1681 cm^−1^) can be also assigned to β-turns (Bock and Damodaran 2013).

In the case of field bean, formula and improved formula a positive band with maximum between 1691 and 1697 cm^−1^ characteristic to anti-parallel β-sheet (Kong and Yu 2007) appears accompanying the increase of band characterizing β-sheet. As the band with maximum at 1629 cm^−1^ is relatively broad one can conclude that pseudo β-sheet can be formed at the expense of β-turns, which indicates production of more structuralized protein pattern at all non-gluten doughs. The hydration of proteins leads to the replacement of protein–protein interactions by protein–water interactions allowing the movement of the polypeptide chains to form β-sheet structure (Correa et al. 2014). This high ability of gluten-free doughs to form β-sheet structure could be explained by their high water absorption. Previous studies evaluating the effect of addition of dietary fibre on the rheological behaviour and structure of gluten proteins (Nawrocka et al. 2016, 2017) showed a strong structuralization of gluten network by forming of β-type structures especially β-sheets. This confirms our results, which implies that the high structuralization of gluten-free doughs by the formation of β-sheet structure may result in their solid behaviour and their low elasticity.

To estimate the effect of the supplementation of rice flour with that of field bean, and to determine the effect of hydrothermal treatment on non-gluten protein conformation, changes in the secondary structure of non-gluten proteins were studied. As rice spectrum in the amide I region varied from other spectra, differential curves were analysed against rice dough protein (Fig. 3b). Rice dough protein contains more aggregates and pseudo β-sheet (positive band at c.a. 1600 cm^−1^ and at c.a. 1615 cm^−1^), but less β-turn (negative band at c.a. 1683 cm^−1^) than other doughs (Fig. 3b A–C). Therefore, one can conclude that rice dough proteins are more structuralized than that of field bean, formula and improved formula doughs.

When we examine the effect of hydrothermal treatment, the process of the change in protein structure seems to be more complex. Generally, the increase of β-sheet (1630 cm^−1^) is observed at the expense of β-turns (1662 cm^−1^) and aggregated structures (1596 cm^−1^). Interesting is the presence of a positive band with a maximum at 1648 cm^−1^. IR band between 1640 and 1648 cm^−1^ is usually associated with the unordered conformation (random coil) (Kong and Yu 2007). As this band is relatively broad, it can also indicate a slight increase in the α-helix fraction, thus confirming the deconvolution analysis. The presence of two positive peaks at 1630 and 1648 cm^−1^ indicates possible structural changes in β-sheet (1630 cm^−1^), a possible increase in the unordered or α-helix fraction (positive peak at 1648 cm^−1^ accompanied with a negative band centred at 1662 cm^−1^) at the expense of aggregates (negative band at 1596 cm^−1^) and pseudo β-sheet (negative band at 1616 cm^−1^).

### Analysis of starch conformation

FT-IR spectra in the region between 945 and 1195 cm^−1^ (Fig. 3S, Supplementary materials) were used to obtain information about the starch structure (Meziani et al. 2011). This part of spectrum represents COH bending and CH_2_ related modes (1077, 1047, 1022 and 994 cm^−1^) and CO and C–C stretching modes (1150 cm^−1^). Peak at 994 cm^−1^ is sensitive to water content and starch conformation (van Soest et al. 1995).

Absorbance bands at 1047 cm^−1^ and 1022 cm^−1^ are characteristic of crystalline and amorphous fractions of starch, respectively. The absorbance intensity ratio R equal to I (1047 cm^−1^)/I (1022 cm^−1^) is frequently used as an indicator of the organization of different types of starch components (Hernández-Uribe et al. 2010; Smits et al. 1998; Meziani et al. 2011). High value of this ratio shows domination of the crystalline conformation over the amorphous conformation which in turn indicates that starch has a strong tendency to retrogradation (Smits et al. 1998).

The highest value of R ratio was noted for field bean dough (0.80) and the lowest for rice dough (0.60). It amounted to 0.67 for wheat dough, to 0.63 for formula dough and to 0.64 for the improved formula dough (Table 1S, supplementary materials). Comparison between R ratios of doughs showed a significant difference between crystalline I (1047 cm^−1^)/amorphous I (1022 cm^−1^) ratios of doughs except between those of formula and improved formula dough. Field bean dough had a highest value of this ratio. Such finding indicates that field bean starch has a high ability to organize in a crystal form by retrogradation (high retrogradation tendency) (Hernández-Uribe et al. 2010).

In the case of field bean dough, starch spectrum shows a major difference in shape and is less intense in comparison with spectra registered for other doughs. Locations of the bands for field bean dough are shifted approximately by 4 cm^−1^ as compared to other spectra. Bands characteristic for starch in doughs show a maximum absorbance at ~ 995 cm^−1^ for field bean, ~ 997 cm^−1^ for soft wheat, and at ~ 1016 cm^−1^ for rice. Formula and improved formula doughs spectra have maximum absorbance at the same position (~ 1014 cm^−1^). Shift of maximum absorbance of starch band for field bean dough to lower wavenumbers can suggest that the starch components of field bean dough are characterized by longer bonds, whereas those of the other doughs have shorter bonds resulting in higher wavenumbers. This phenomenon is probably connected to a botanical origin of flour components containing different types of starches (Cai et al. 2014).

### Relationship between structural and rheological parameters of doughs

Table 3 shows the correlation matrix between rheological and structural parameters of doughs obtained in this study. Strong and positive correlations were observed between β-sheet and α-helix content with TDD. On the other hand, the latter parameter showed an inversely proportional relationship with β-turn content (negative correlation). These observations may indicate that a long TDD that characterized gluten-free dough promoted the formation of β-sheet and α-helix structures in the detriment of β-turn structure. This finding confirmed the results obtained from the analysis of the differential spectra. Our results are in line with those obtained by Nawrocka et al. (2016) that showed a positive correlation between dough developing time and the content in α-helix and β-type structures and negative with the β-turn content. In parallel, tan δ was strongly and positively correlated with the β-turn content, but negatively correlated with the α-helix and β-sheet content. This could indicate that the high structuralization level is realized by the strong tendency of gluten-free doughs to form α-helix and β-sheet structures. It explains their low tan δ values and, therefore, the strong participation of the solid character during dough formation. According to the results obtained in previous rheological studies on gluten matrix conducted by Nawrocka et al. (2017) and Nawrocka et al. (2016), the high structuralization level of gluten-free protein backbone was probably connected with the strong and stiff behaviour of gluten-free doughs compared to that of soft wheat.

**Table 3:** Correlation matrix between the rheological parameters [WA, TDD, G′, G″, tan (δ)], doughs proteins and starch structural parameters

Variables	WA	TDD	G′	G″	Tan (δ)	β-Sheet	α-Helix	β-Turn
TDD	0.409							
G′	0.608	0.376						
G″	− 0.149	− 0.117	0.669					
Tan (δ)	− 0.642	− 0.664	0.035	0.742				
β-Sheet	0.423	**0.956**	0.151	− 0.395	− 0.825			
α-Helix	0.423	**0.956**	0.151	− 0.395	− 0.825	**1.000**		
β-Turn	− 0.482	− 0.855	− 0.039	0.582	**0.926**	− **0.944**	− **0.944**	
R	− 0.592	− 0.108	0.279	0.850	0.803	− 0.355	− 0.355	0.527

R: The ratio I (1047 cm^−1^)/I (1022 cm^−1^)

Values in bold are different from 0 to a significance level α = 0.05

The R ratio was positively correlated with loss modulus and loss tangent of doughs. These parameters were probably affected by the reorganization of doughs’ starches. Initially, low values for rice dough increased upon the supplementation with field bean or with the application of hydrothermal treatment, indicating the tendency of starch to retrogradation. This result may explain the highest value of viscous modules of field bean dough.

## Conclusion

Food industry can benefit from good understanding of structure formation and its connection with rheological properties of non-gluten doughs. A link between structure and rheological properties was established. This study clearly demonstrated that gluten-free doughs, with the exception of field bean dough, behave like a rigid and stiff material with a low elasticity compared to soft wheat dough. Supplementation with rice-field bean significantly changed the rheological behaviour of doughs by increasing the loss modulus to be similar to that of wheat dough. At the structural scale, with respect to proteins forming soft wheat dough, all gluten-free doughs shown a tendency to form β-sheet structure in detriment of β-turn structure. These doughs show higher level of protein network structuralization as compared to soft wheat dough. This could be the reason for the low level of gluten-free dough elasticity. The improvement of rheological properties of the rice dough after supplementation was accompanied by a less structured protein network of formula dough (decrease of β-sheet and α-helix contents). Hydrothermal treatment induced the formation of more ordered structures such as β-sheet and α-helix. Rice-field bean supplementation and hydrothermal treatment induce positive changes in the starch conformation approaching that of wheat dough.

## Electronic supplementary material

Below is the link to the electronic supplementary material.

Supplementary material 1 (DOCX 199 kb)

## Abbreviations

ATR: Attenuated total reflectance
FT-IR: Fourier transformed infrared spectroscopy
FU: Farinograph unit
I: Intensity
TDD: Time of dough development
WA: Water absorption
