# Supplementary material for: Investigation of viscoelastic behaviour of rice-field bean gluten-free dough using the biophysical characterization of proteins and starch: a FT-IR study
Source: J Food Sci Technol. 2019 Feb 22;56(3):1316–27. doi: 10.1007/s13197-019-03602-2 (PMC6423174; doi:10.1007/s13197-019-03602-2)
Supplement: Supplementary file 1 — Supplementary material 1 (DOCX 199 kb) [file 13197_2019_3602_MOESM1_ESM.docx]

Fig 1S. Variation of rheological parameters of doughs as a function of frequency; A storage modulus (G’), B loss modulus (G”) and tan (δ).

**
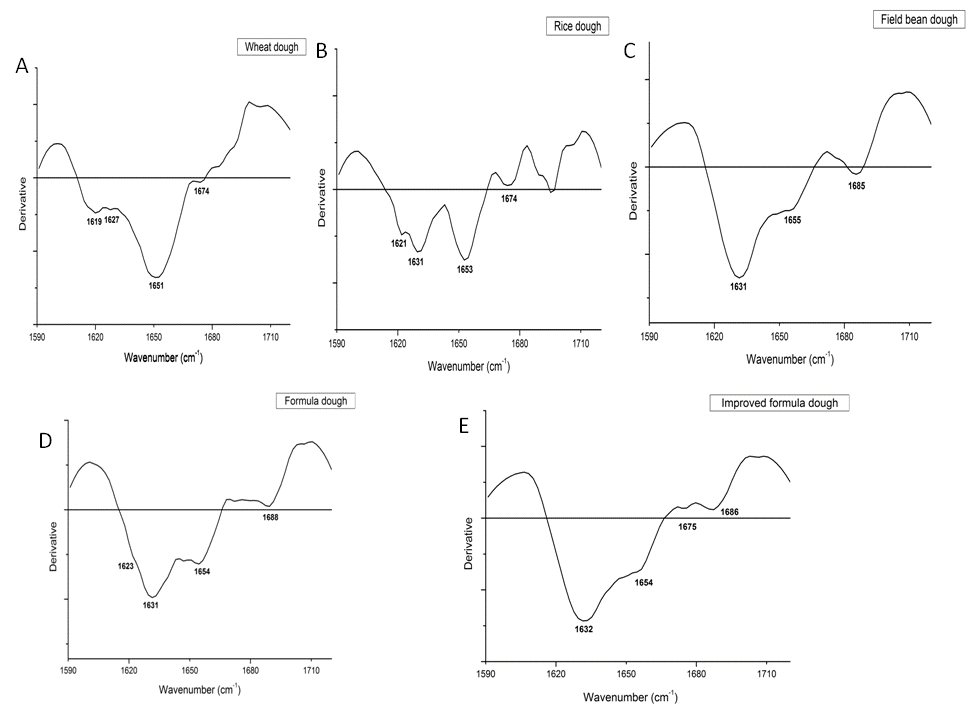
**

**Fig. 2S** Second derivative of FTIR spectra of doughs in amide I region (1590-1720) cm^-1^; (A) soft wheat dough, (B) rice dough, (C) field bean dough, (D) formula rice-filed bean dough and (E) improved formula dough. 2^nd^ derivative was calculated using five points two-degree polynomial function and smoothed with 11-points two-degree polynomial Savitsky-Goly function.


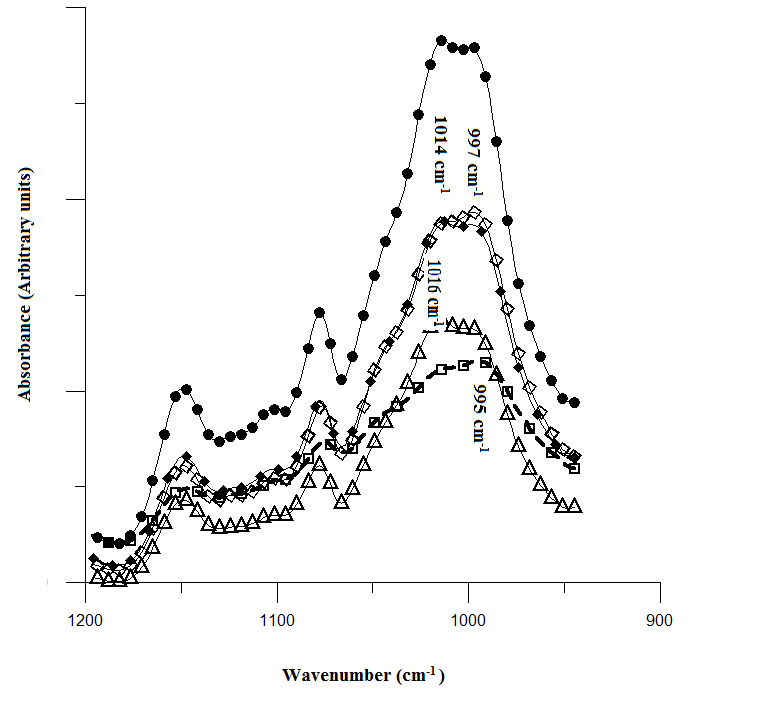


**Fig. 3S.** Spectral region of starch (945-1195) cm^-1^ registered for wheat (*diamond*), rice (*open triangles*), field bean (*open squares*), formula (full *diamonds*) and improved formula (*dots*) doughs. 1077, 1047, 1022 and 994 cm^-1^: COH bending and CH_2_ related modes , 1150 cm^-^1: CO and C-C stretching modes, peak at c.a. 995 cm^-1^ : water content and starch conformation.

Table 1S.

R values of ratio between crystalline (I (1047cm^-1^)) and amorphous (I (1022 cm^-1^)) conformations of doughs

| Doughs | R value |
| --- | --- |
| Wheat | 0.67 |
| Rice | 0.60 |
| Field bean | 0.80 |
| Formula | 0.63 |
| Improved formula | 0.64 |

 R values were obtained by calculation of absorbance intensity ratio between intensities of crystalline conformation at 1047 cm^-1^ and amorphous conformation at1022 cm^-1^.
